# Virtual Faculty Development in Simulation in Sub-Saharan Africa: A Pilot Training for Pediatricians in Kisumu, Kenya

**DOI:** 10.3389/fped.2022.957386

**Published:** 2022-09-23

**Authors:** Colleen Fant, Macrine Olwala, Grace M. Laanoi, Gatwiri Murithi, Walter Otieno, Elizabeth Groothuis, Ashti Doobay Persaud

**Affiliations:** ^1^Division of Emergency Medicine, Department of Pediatrics, Ann and Robert H. Lurie Children's Hospital of Chicago, Chicago, IL, United States; ^2^Center for Global Health Education, Feinberg School of Medicine, Havey Institute for Global Health, Northwestern University, Chicago, IL, United States; ^3^Jaramogi Oginga Odinga Teaching and Referral Hospital, Kisumu, Kenya; ^4^Maseno University School of Medicine, Kisumu, Kenya; ^5^Center for Public Health and Development, Nairobi, Kenya; ^6^Division of Hospital-Based Medicine, Department of Pediatrics, Ann and Robert H. Lurie Children's Hospital of Chicago, Chicago, IL, United States; ^7^Departments of Medicine and Medical Education, Feinberg School of Medicine, Northwestern University, Chicago, IL, United States

**Keywords:** simulation based medical education (SBME), resource limited settings, Kenya, faculty development, virtual education, global health

## Abstract

**Introduction:**

Simulation is an effective educational tool increasingly being utilized in medical education globally and across East Africa. Globally, pediatric patients often present with low frequency, high acuity disease and simulation-based training in pediatric emergencies can equip physicians with the skills to recognize and intervene. Northwestern University (NU) in Chicago, IL, USA, and Maseno University (MU), in Kisumu, Kenya launched a predominantly virtual partnership in 2020 to utilize the Jaramogi Oginga Odinga Teaching & Referral Hospital (JOOTRH) simulation center for MU faculty development in simulation based medical education (SBME) for medical students.

**Materials and methods:**

Educational goals, learning objectives, and educational content were collaboratively developed between MU and NU faculty. Virtual sessions were held for didactic education on simulation pedagogy, case development, and debriefing. Mixed educational methods were used including virtual mentored sessions for deliberate practice, piloted case facilitation with medical students, and mentored development of MU identified cases. Trained faculty had the summative experience of an intensive simulation facilitation with graduating MU students. MU faculty and students were surveyed on their experiences with SBME and MU faculty were scored on facilitation technique with a validated tool.

**Results:**

There were four didactic sessions during the training. Seven cases were developed to reflect targeted educational content for MU students. Six virtually mentored sessions were held to pilot SBME with MU students. In July 2021, fifty students participated in a week-long SBME course led by the MU trained faculty with virtual observation and mentorship from NU faculty. MU faculty reported positive experience with the SBME training and demonstrated improvement in debriefing skills after the training. The overwhelming majority of MU students reported positive experiences with SBME and endorsed desire for earlier and additional sessions.

**Discussion and conclusions:**

This medical education partnership, developed through virtual sessions, culminated in the implementation of an independently run simulation course by three trained MU faculty. SBME is an important educational tool and faculty in a resource constrained setting were successfully, virtually trained in its implementation and through collaborative planning, became a unique tool to address gaps for medical students.

## Introduction

Global healthcare inequities are multifactorial, and contributing factors include a paucity of practitioners as well as limited access to specialty training (1). Simulation-based medical education (SBME) can be used to address healthcare inequities by allowing medical students and general practitioners to gain skills in specialized patient scenarios they may not typically (or frequently) encounter in their training. For example, simulation training has been shown to improve learners' abilities to manage emergencies (2, 3). In many parts of the world including sub-Saharan Africa, where medical education has historically relied heavily on didactics followed by direct patient care (4), SBME allows for a safe transition between the classroom and the hospital as it can be used to expose trainees to situations that they may not otherwise encounter without putting patients at risk (5). SBME, however, remains in its early stages in sub-Saharan Africa.

Historically, simulation has been thought of as taking place solely in high-fidelity labs with costly technology, making it difficult to implement in resource-limited settings. Successful implementation of SBME in a variety of educational settings, including those with fewer resources, has demonstrated that this is not true. In fact, simulation is quite well-suited to teaching with a variety of resources and can be done *in-situ* (in locations of patient care), with low-fidelity tools, and even virtually. SBME has been implemented in these various forms, including in high-fidelity labs, across the African continent. This educational strategy has been used to train learners on surgical skills in Ghana, Tanzania, Ethiopia, Zambia, Uganda, Kenya, and other countries over the last several decades (6). In Rwanda, a simulation center was established in partnership with a Canadian institution that was utilized by trained local faculty across disciplines (7). The Vital Anesthesia Simulation Training (VAST) course has been used to train anesthetists in Rwanda, Tanzania, Ethiopia, India, Fiji, and Canada on essential non-technical skills (8).

There are three phases of a successful simulation: the case pre-brief, the facilitation of the case, and the case debrief. Faculty educators using simulation must learn how to appropriately develop and select cases, as well as master each of the simulation phases (9, 10). Learning is active and self-paced during simulation; learners apply theory to practice, and self-critique their thought processes and actions. Additionally, learners get real-time feedback from expert faculty during cases to identify areas of improvement and reinforce best practices. Simulation can be used to address a diverse range of topics including clinical care, technical skills, effective communication, and clinical decision-making.

Simulation is particularly useful in pediatrics, where emergencies are less common but can be clinically challenging. SBME allows the learner to practice emergency management skills without causing patient harm when mistakes are made. One Kenya-based study identified that much of inpatient pediatric mortality occurs early in hospitalization (11). In another study, Kenyan providers found their interns ill-prepared for patient care upon graduation from medical school (12). These two studies highlight the need for better trainee preparation and support the use of training aimed specifically at the early recognition of critically ill pediatric patients and their subsequent resuscitation. At Maseno University (MU) in Kisumu, Kenya, clinical education for medical students is largely comprised of didactic lectures, teaching on rounds, case discussions/tutorials, and exams for assessment. In 2017, a new simulation lab was established at Jaramogi Oginga Odinga Teaching and Referral Hospital (JOOTRH), the primary teaching hospital for MU. The simulation lab has been used for clinical training in various specialties including anesthesia, obstetrics, and more recently, management of critically ill patients with COVID-19. The COVID-19 pandemic decreased interaction between medical students and patients and created the need to use alternative tools to provide clinical training in a safer environment. A study looking at data from 204 hospitals found a decline in admissions under the age of 5 for medical and surgical care (13), further emphasizing the role of SBME in pediatric training. To address this need for medical students rotating in pediatrics at MU, an online SBME faculty development curriculum was co-created *via* a collaboration between Northwestern University Feinberg's School of Medicine's Center for Global Health Education (CGHE) and MU. The curricular goal was to teach simulation and debriefing pedagogy and skills to pediatric faculty educators to use with their medical learners.

## Materials and methods

### Curriculum development

An online SBME faculty development curriculum was developed in 2020 and then implemented with MU faculty in the department of pediatrics between October 2020 and July 2021. A team from MU, JOOTRH and NU's CGHE was assembled, and a targeted needs assessment was completed through strategic planning sessions. The sessions explored MU faculty as learners (experiences, preferences, expectations, etc.), stakeholder needs, facilitators, resources, and barriers. The team reviewed existing curriculum from NU, and together they used available resources, demography of patients seen at JOOTRH, and educational gaps to guide the selection of educational content and instructional strategies. Pediatric emergency cases were identified through this process and thus targeted for initial case development. The PEARLS (Promoting Excellence and Reflective Learning in Simulation) framework was used to guide debriefing (14). The goal of the curriculum was to train MU faculty in the facilitation of SBME, so that they could independently design and implement a simulation program for medical students during their pediatric rotations at JOOTRH. Brief, intermittent, mixed methods online synchronous educational sessions were used to introduce topics and build SBME mastery in facilitation.

### Curriculum implementation

Sessions were held over 12 months using an online collaborative platform, with MU faculty located at the high-fidelity simulation center at JOOTRH, and NUCGHE faculty teaching and facilitating remotely. Introductory content was delivered through didactic sessions that were interspersed with demonstrative videos. Collaborative case development was done with MU and NUCGHE faculty over email and through virtual meetings to reflect the context at JOOTRH and match resources in the simulation lab. Trainings were done entirely virtually, and four educational methods were used: didactic training, internal practice, observed deliberate practice, and ultimately a summative simulation session run independently by learners. Deliberate practice was first done within the group of faculty learners to familiarize themselves with SBME facilitation; practice then continued with mentored sessions in which participating MU faculty facilitated cases in person for medical students, and NUCGHE faculty were available virtually. Directive feedback was provided as faculty practiced simulation debriefing, and self-assessment was encouraged reflecting domains covered in a validated debriefing rubric—the Objective Structured Assessment in Debriefing (OSAD) tool (15, 16). Throughout the sessions, educational and case content was adjusted to reflect learner-driven goals. A capstone experience was supported with MU faculty learners leading a simulation symposium for medical students.

### Evaluation

Several strategies were used to evaluate the curriculum implementation. Learner perceptions and feedback were self-reported *via* survey halfway through the curriculum. Additionally, debriefing skills were targeted as a primary assessment outcome and were evaluated *via* the OSAD rubric on initial and final debriefing experiences (15). Assessments were completed by curriculum leadership, and learners were also invited to self-assess after viewing recorded sessions in order to identify personal areas of improvement in the domains of Approaching, Environment, Engagement, Reaction, Reflection, Analysis, Diagnosis, and Application (15). Finally, during the summative experience, the participating medical students at JOOTRH were anonymously surveyed about their experiences and satisfaction.

## Results

The virtual faculty development curriculum for SBME is described below (Table 1). Using this curriculum, three MU pediatric faculty were trained in SBME to facilitate cases in pediatric emergencies for medical students rotating through pediatrics. There were six deliberate practice sessions to build SBME skills. Seven clinical cases were developed in collaboration between NU and the MU faculty to address identified educational gaps for MU students, reflecting the clinical experience at JOOTRH (Table 2). As a final component of the curriculum, MU faculty hosted a 5-day simulation symposium for all fifty graduating MU medical students.

**Table 1:** Virtual faculty development curriculum.

***Curricular goals***
To work with pediatric faculty at Maseno University and provide them with a comprehensive training in an educational tool, pediatric simulation, for them to use in teaching their trainees to care for acutely ill children.
***Curricular objectives***
1. By the completion of this training, the participants will be able to describe and apply the basic pedagogical framework behind simulation in medical education 2. By the completion of this training, the participants will be able to describe and apply the fundamentals of the PEARLS framework for simulation debriefing 3. By the completion of this training, participants will lead and debrief a remote simulation case and receive constructive feedback from their peers 4. By the completion of this training, participants will have the tools to develop, run, and debrief pediatric simulation cases for medical trainees
***Curricular activities***
**Phase of curriculum**	**Educational method**	**Educational content**
Introduction to Simulation	Didactic Sessions	***Lecture***: Introduction to Simulation ***Lecture***: Introduction to Debriefing—the PEARLS framework ***Lecture***: Pedagogy of Simulation for Medical Education ***Lecture***: Writing Learning Objectives for SBME ***Workshop***: Developing a Simulation Case ***Lecture***: Different Learner Types, Troubleshooting Facilitation, and Incorporating Communication into the Case
Internal Deliberate Practice	Virtual Simulation Case Facilitation	Faculty learners practiced taking on roles of learner, observer, and facilitator within their learner group and would repeat case facilitator roles as needed
	Virtual Simulation Case Facilitation—Developing a New Case	Faculty learners practiced taking on roles of learner, observer, and facilitator within their learner group for a case they developed
External Deliberate Practice	Virtual Simulation Case Facilitation with NU virtual Mentorship	Faculty learners had observed facilitation of 7 cases with virtual mentorship from NU Simulation faculty with direct feedback. Rotating medical students participated as pilot participants.
Cumulative Experience	Five-day simulation symposium held for medical students	50 medical students participated in simulation facilitated by trained faculty over an intense 5-day period. NU faculty present virtually to assess performance and provide feedback. Every student participated in or observed a neonatal resuscitation case and two others.

**Table 2:** Clinical cases for simulation.

**Clinical cases**
Neonatal Resuscitation Neonatal Sepsis Respiratory Distress Syndrome Neonatal Jaundice Hypovolemic Shock from Diarrheal Disease Pneumonia/COVID Pneumonia Ingestion (Organophosphate Poisoning)

**Figure 1:**
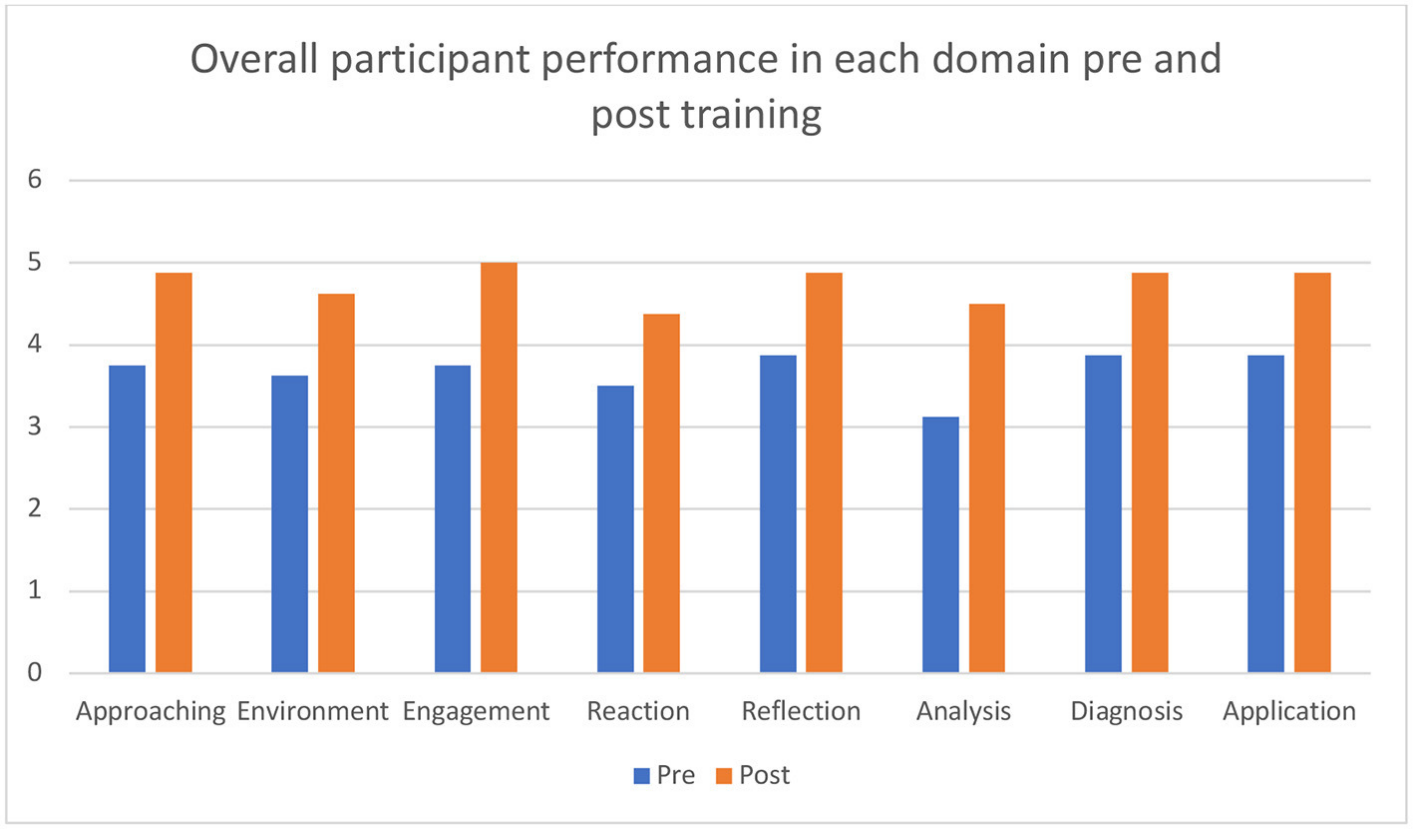
Average OSAD scores across all domains before and after facilitator debriefing training.

MU faculty learners were surveyed online and reported little prior exposure to simulation and reported positive experiences with the curriculum. Two NU faculty assigned OSAD scores to primary and final observed case facilitation, and faculty learners were invited to self-assess as well. Skills were shown to improve from first to last debriefing session when averaging scores among all participants (Figure 1).

Medical students participating in the simulations overwhelmingly reported positive experiences *via* anonymous online survey accessed *via* QR codes. Thirty-five of the fifty students completed the online survey and reported that participating in simulation was useful and valuable, and would recommend it to their peers. All either partially or fully agreed that they were more confident taking care of children after participation in simulation. In a free-text portion of their evaluation, student comments included a request for increased frequency of simulation cases, for earlier incorporation of simulation into their education, and for simulation to be included in other disciplines.

Additionally, one of the pediatric faculty learners who completed the curriculum presented the simulation training program at two international conferences.

## Discussion

Through this innovative partnership, three pediatric faculty were successfully trained in simulation-based medical education using a novel curriculum. Medical students reported a high degree of satisfaction with the simulation sessions run by MU faculty.

The rise of virtual meeting platforms during the COVID-19 pandemic allowed the development of a relationship between NUCGHE and MU faculty in a way that would not have otherwise been possible due to restrictions on international travel. By establishing an ongoing virtual partnership, it became possible to collaborate on educational content, goals, and curricular feedback without relying on in-person sessions. The partnership between NUCGHE and MU/JOOTRH developed quickly as busy faculty from both NU and MU held brief virtual meetings at mutually convenient times. Furthermore, by conducting all training sessions online, the mentorship was able to be longitudinal, intermittent, and adaptive to learner needs.

This partnership and curriculum were developed at a key time, as there was a desire to increase the utilization of the high-tech, high fidelity JOOTRH simulation lab. However, its ability to be tailored to individual audiences suggests that this curriculum could also be implemented in variable contexts and potentially lower-tech environments. In educational settings without advanced simulation technology, more emphasis could be placed on *in-situ* simulation; if the simulations are performed in an empty patient room, there need not be a dedicated simulation lab. Additionally, simulations can easily be run with low-fidelity mannequin with simple case adaptations.

There were several limitations of this program. As it was limited to a single institution, only three pediatric faculty were trained in the curriculum; however, these three faculty members are responsible for the bulk of the medical student education in pediatrics at JOOTRH. Time commitment is often a challenge in educational efforts for busy and often overworked medical providers. The virtual, intermittent model helped mitigate this by spreading out content across multiple meetings using spaced repetition of key concepts. Sessions were limited to 2 h and were held at a times most convenient to both learners and facilitators.

One of the most rewarding outcomes of this program was the ability to not only to have three trained pediatric faculty independently implement simulation for pediatric emergencies for medical students, but to also cultivate a medical education partnership through which additional collaborations are evolving. Through this partnership, MU faculty was mentored through a first international conference presentation and NU faculty collaborated with the MU simulation center faculty on a book chapter and a manuscript. The partnership between NUCGHE and MU continues with training in advanced simulation skills, such as rapid cycle deliberate practice. Faculty also continue to collaborate on the development of additional pediatric simulation cases. In the next phase of this partnership, the curriculum has been adapted to be specialty-agnostic, expanding the faculty development program to several other specialties at MU/JOOTRH.

## Conclusions

This curriculum represents one model of a virtually implemented faculty development curriculum to improve local capacity of SBME at a medical school in Kisumu, Kenya. Three pediatric faculty continue to facilitate simulations for pediatric emergencies for rotating medical students and have developed an initial case library to use with their students. Trained faculty demonstrated high scores on their case debriefing skills and medical students benefitting from this newly implemented educational tool reported overwhelmingly positive experiences, clamoring for more.

## Data availability statement

The raw data supporting the conclusions of this article will be made available by the authors, without undue reservation.

## Author contributions

CF led and implemented the curriculum and wrote the primary manuscript. MO, GL, WO, EG, and AD assisted in curriculum content and manuscript preparation. All authors contributed to the article and approved the submitted version.

## Funding

This program was generously supported by the Strohm Family Grant and the Robert J. Havey, MD Institute for Global Health's catalyzer fund at Northwestern University, Feinberg School of Medicine.

## Conflict of interest

The authors declare that the research was conducted in the absence of any commercial or financial relationships that could be construed as a potential conflict of interest.

## Publisher's note

All claims expressed in this article are solely those of the authors and do not necessarily represent those of their affiliated organizations, or those of the publisher, the editors and the reviewers. Any product that may be evaluated in this article, or claim that may be made by its manufacturer, is not guaranteed or endorsed by the publisher.
